# Immunotherapy as a treatment to confront the ongoing opioid epidemic- A review

**DOI:** 10.46439/immunol.1.006

**Published:** 2022

**Authors:** Nachum Dafny

**Affiliations:** UT Houston, McGovern Medical School, Department of Neurobiology and Anatomy, Houston Texas 77030 USA

**Keywords:** Morphine, Addiction, Interferon, Cyclosporin, Cortisol

## Abstract

Substance use disorders continue to be major medical and social problems worldwide. The use of opiate has grown substantially over the past three decades reaching the dimensions of a global epidemic. Current drug treatments have many limitations: long treatment times, dependency on treatment medications, relapses after treatment, high costs of treatment, and non-adherence by affected persons. Most of the available drug treatments for opiate addiction belong to the opioid family. Some worry that the availability of the drugs may simply cause substituting one opioid medication for another. Immunotherapy has a great potential of becoming an additional therapeutic strategy in the treatment of addiction. Immunotherapy also prevents overdose of treatment drugs. This monograph reviews preclinical studies of immunotherapy and experiments using treatments with three different immunomodifiers that were able to significantly attenuate the severity of opioid withdrawal symptoms in morphine dependent animals. These immunotherapy treatments are short, and will prevent relapse of opioid dependency and toxicity.

## Introduction

The use of opiate for the treatment of pain has escalated in recent years making them one of the most commonly abused prescribed medicines in the U.S.A. [1]. Opiate addiction is a chronic brain disease. Most preclinical and clinical treatments for opiate addiction involve long term treatment with medications such as: Zubsolve, Probuphrine, Lofexidine hydrochloride, Methadone, Buprenorphine, Sublocade, CAM2038, Naltrexone, Pentazocine, Buprenex, Modafinil, Mirtazapine, Vigabatrin, Baclofen, and Topiramate [2]. The efficacy and safety of most of these drugs have not been adequately studied [3]. Counseling and behavioral therapies and/or family and spiritual support for long periods of time are essential along with these drug therapies. As most of the above drugs belong to the opioid family, some worry that the above treatments are substituting one opioid medication for another opioid [3]. Furthermore, most patients do not comply with these long-term treatments. Most of these drugs are for opiate withdrawal management, and results show that they lead to stronger cravings and relapses which limits overall effectiveness. The available treatment drugs simply substitute one opioid for another, and this may prolong a type of “dependency”. Therefore, it is essential to study other means to treat addiction.

### The Current Opioid Crisis (Epidemiology)

Since 1980 the overdose death rate from opioid consumption in America has increased exponentially at a rate of 76% per year, and currently reaching 50,000 deaths per year. Pitt et al. [4], estimate that on the current course, just over half a million Americans will die of opiate overdose from 2016 to 2025. The current opioid epidemic began in the 1990s with over-prescription of opioids as pain relieving medications. Opioids quickly became the most prescribed medication class in the USA. About 20 to 30 percent of patients, who are prescribed opioids for chronic pain misuse them [1]. About 80 percent of heroin users first misuse prescription opioids. In the same year about 4.3 million peoples used opiates for nonmedical purposes. There are more opioid overdose deaths in the US every year than deaths due to car accidents and gunshots together [5]. During a 12-month period ending in Nov. 2017, 69,948 lives nationwide were lost due to opioid overdose making it one of the most serious overdose crises the country has ever had to face [6]. More than 210 million opiate prescriptions were filled in 2010 with close to 12 million people admitting to abusing these drugs by taking them for non-medical reasons. Misuse of prescription opioids affects millions of Americans. In 2015, an estimated 20,100 deaths were due to prescription painkillers and 12,940 deaths were due to heroin use. In the same year, 591,000 people had a substance abuse disorder [7]. In 2016 and 2017, more than 42,000 and 47,600 deaths in the US were caused by overdose of prescription opioids respectively [1,8]. Another paper reports that more than 70,000 fatalities, or approximately 200 deaths per day, occurred in 2017 [9]. Over opioid prescription continues to be national epidemic [3]. As the opioid crisis continues to devastate the USA, it is essential to investigate new approaches to combat this crisis [3]. A new approach is the objective of this review.

### Morphine

Morphine is a pain medication of the opiate family found in plants and animals. In clinical settings, morphine exerts its principal pharmacological effect on the central nervous system (CNS) as a pain reliever. It may also be used as a sleep aid or a treatment for gastro intestinal track spasms. Morphine binds to its receptors and triggers chains of biochemical events that modulate the neuronal activities of many brain areas that regulate behavior. This manuscript aims to review studies of effective short-term non-opioid immunomodifier treatments to prevent and treat morphine tolerance and withdrawal.

### Opiate Addiction and Glial cells

The CNS is built from two broad categories of cells, neurons and glial. The glial cells outnumber the neurons and the two cell types occupy a comparable amount of space in brain [10,11]. The glial system was considered to be a passive accessory to neurons. It has been demonstrated that these cells actively participate in synaptogenesis, neuronal excitability, and neurotransmission [11]. Glial cells also maintain an appropriate concentration of ions and neurotransmitters in the neuronal environment. An increasing body of evidence indicates that the glial cells are essential regulators of the formation, maintenance and function of synapses, the key functional unit of the CNS [11]. Moreover, the glial system exhibits robust synaptic plasticity as well as changes in its morphology and physiology in response to opiate exposure within key brain sites contributing to addiction [12,13]. Synaptic plasticity consists of changes in synaptic strength that is believed to be the basis of learning and memory. For a very long period of time synaptic plasticity has been a hallmark of neurons. Recent advances in the physiology of glial cells indicate that they possess all the features necessary to modulate the various forms of synaptic plasticity. Indeed, beside their respective supportive and immunologic functions, an increasing number of studies demonstrate that glial cells express receptors for most neurotransmitters and release neuroactive substances that have been shown to modulate neuronal activity, i.e., they express synaptic plasticity. Because glial cells surround neurons and their synapses and release a wide variety of neuroactive molecules during physiological and pathological conditions, they have been shown to modulate synaptic plasticity in many different ways from changes in synaptic coverage to release of chemokines and cytokines [10,14]. Glial cells are considered to be part of the immune system. Glial cells are nomadic immune cells of the CNS and active participants in the generation of innate immune response. They are considered to be the guards within the CNS due to their complication to Toll-like receptors [15]. The Toll-like receptor 4 is a potential site for opioid-induced glial activation [10,16]. Accumulated evidence indicates that the glial cells play an essential role in modulation of the opioid reward neuronal circuitry and participate in opioid-elicited addiction [16].

It has been reported that acute and chronic morphine and psychostimulant use activate specific components of the innate immune system [15]. Opiates activate brain microglia to release various factors, which in turn contribute to opiate tolerance, dependent and withdrawal symptoms, based on these Evans and Cahill [13] and Venkataraman and Dafny [17] suggest that repeated opiate consumption induce adaptive changes that modify neuronal circuitry and create an altered “normality” - the “drug dependent” state. The neuron adapts in many ways to the repeated use of opiate and reaches a new allostatic state- the “drug dependent” state. The involvement of the immune system in the response to opiates has developed to such an extent that immunotherapy is being considered in the management of addiction [15,17]. It has been suggested that the effects of drug abuse on the immune cells in the brain can be summarized in three steps. 1^st^: action on glial cells to generate the production and release of proinflammatory cytokines. 2^nd^: cytokines induce activation of quiescent astrocytes and microglia which in turn enhance the inflammatory response. 3^rd^: significant pathways that are initiated by these cytokines activate the immune cells resulting in modulation of brain function [15].

### Morphine and the Immune System

Morphine affect the brain activity, behavior and numerous body system whose cells carry opiate receptors including the immune system [18]. The capacity of the immune system to participate in the processes primarily considered to be brain phenomena has been suggested by several studies that demonstrate that an intact immune system is essential to the expression of opiate withdrawal precipitated by injecting naloxone (Nal) into opiate-dependent animals. It has long been appreciated that opioids such as morphine, exhibit anti-inflammatory activity. This led early on to the speculation that cells of the CNS influence the immune system [19], and that drug withdrawal elicits immune responses that contribution to the development of withdrawal symptoms and relapse [20]. When the immune system is destroyed by selective doses of irradiation, and a subject is then treated with chronic morphine, Nal administration fails to precipitate the expected withdrawal behavior. Moreover, it has been observed that animals that had their immune systems destroyed and then had their immune systems reconstituted by normal donor animals exhibited the expected withdrawal symptoms when they were treated with chronic morphine followed by Nal administration [21,22].

More recently, preclinical studies of immunotherapy show great potential for becoming a new therapy for addiction [23–27]. Two approaches were used: 1) passive immunization that relies on the administration of monoclonal antibodies [25,26], and, 2) active immunization that relies on vaccine injection [27]. Both have great potential to help patients achieve and sustain abstinence for cocaine, nicotine, methamphetamine, and heroin addiction [25–27], but there is a risk of overdose [27]. It seems that immunotherapy may be an additional opportunity for effective treatment of selective drug addicts [27].

Since the discovery of the endogenous opiate, the question arises as to - what prevents the development of tolerance and/or dependence on these endogenous opiates? It was postulated that endogenous production of a protein or a peptide or immune system product such as a cytokine in the central nervous system could prevent the development of tolerance and or physical dependence on these circulating endogenous opiates [28– 30]. Bertolini et al. [31] suggested that some endogenous substances are produced and released along with the endogenous opiates in order to prevent the organism from developing tolerance to or dependence on its own endogenous opiates [32]. Moreover, the endogenous level of alpha- interferon (IFN) is reported to be reduced by repetitive morphine applications [33]. The above was the rationale for a series of experiments to test if immunomodifiers given prior to morphine treatment as well after repetitive morphine treatments i.e., in morphine dependent subjects, will be able to prevent and/or attenuate the severe expression of opiate withdrawal symptoms.

## Results

**Figure 1:**
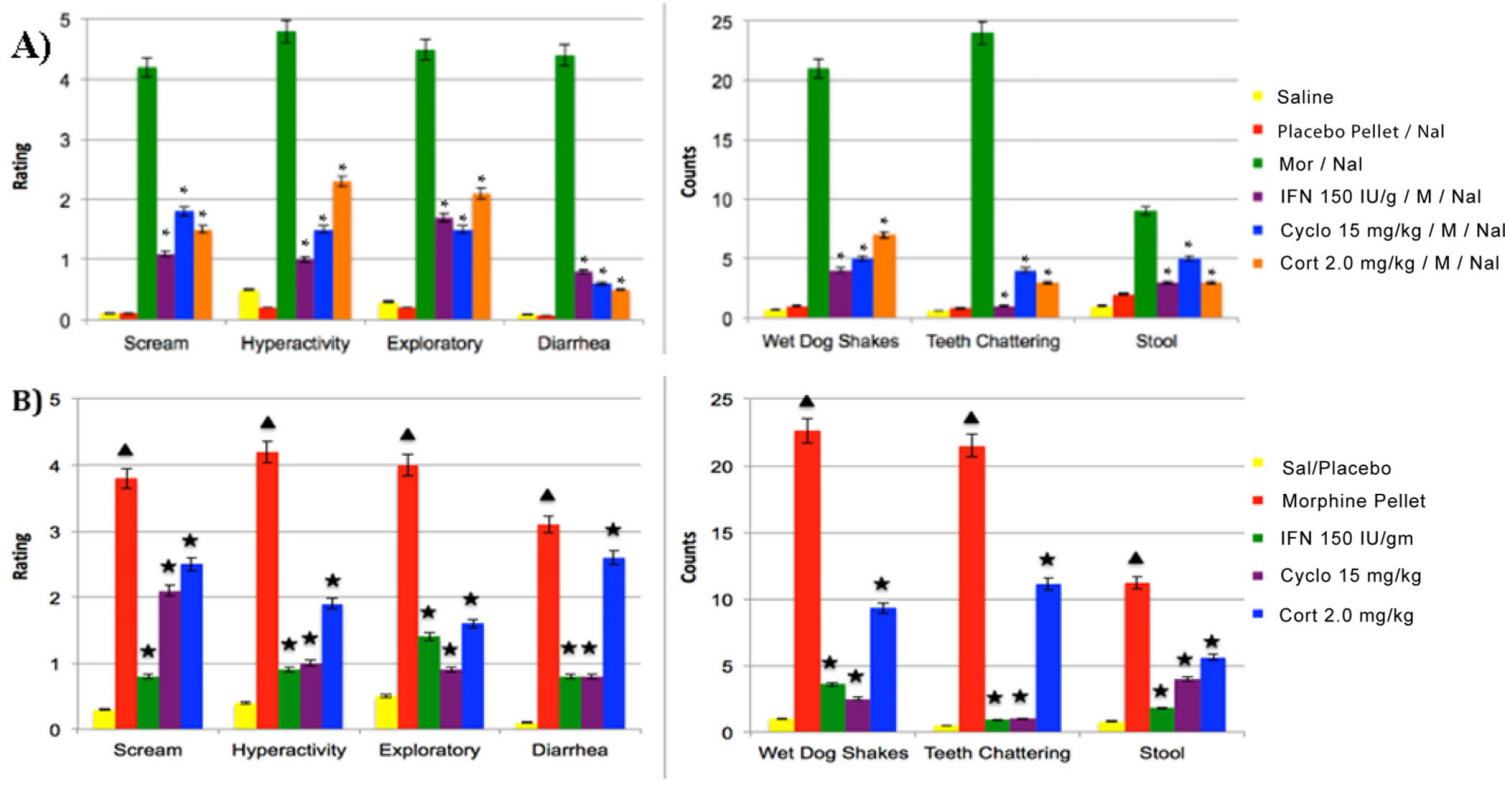
The figure summarizes the behavioral observation of two experiments A and B using Sprague Dawley male rats (Harlem Indianapolis IN USA). The upper part of the Figure 1A summarizes six experimental groups of animals each N=8. Group 1 (yellow column) a control group treated with multiple injection of saline. Group 2 (red column), animals were treated with placebo morphine pellet (as control for morphine pellet), and after 72 hrs. were given naloxone (Nal) 1.0mg/kg. Group 3 (green column) summarizes the observation of the group that was implanted with morphine pellet (75 mg/kg) under the skin and 72 hrs. later the residual morphine pellet was removed and Nal 1.0 mg/kg was given to precipitate withdrawal behaviors. The observation show that Nal injected to morphine dependent animals, increase significantly (p<0.001) all the seven behavioral expression of withdrawals as compared to the control group, i.e., this group expresses the expected behavioral withdrawal symptoms. The next three groups (group 4 – purple, group 5 - blue and group 6 – orange columns respectively) were treated as group 3 (Green), but prior to morphine pellet implantation they were treated either with 150 mg/kg IU/g, IFN - (purple column), or Cyc 15 mg/kg (blue column) or Cort 2.0 mg/kg respectively and one hr. later were implanted with morphine pellet (75 mg/kg). 72 hrs. after morphine pellet implantation the residual morphine pellet was removed and Nal 1.0 mg/kg was given. In all these three groups all the seven behavioral measures were attenuated significantly (p<0.001), i.e., IFN, Cyc, or Cort given prior to chronic morphine attenuated significantly the intensity of the morphine withdrawal behaviors precipitated by Nal treatment. **Figure 1A star sign** – indicate significant (P<0.001) different from Morphine Nal group (green group). Figure 1B present similar experimental groups, the difference is that morphine pellet was implanted first, after 72 hrs. the residual morphine pellet was removed and the animals were treated with IFN, or Cyc or Cort and one hr. later, animals were treated with Nal (1.0 mg/kg) to precipitate behavioral withdrawal. All the three morphine dependent groups that were treated with one of the immunomodifiers prior to Nal injection expresses significant (p<0.001) decrease in the severity of the withdrawal sign expression. **Figure 1B triangle sign** – indicate significant (P<0.001) different from control (yellow group). **Star sign**- indicate significant (p<0.001) different from morphine pellet (red) group. All experimental procedures were approved by the University of Texas Health Science Center Animals Welfare Committee and are in accordance with the National institute of Health of Health Guide for Care Use of Laboratory Animals. **Abbreviations:** Nal: Naloxone; Mor: Morphine; IFN: Interferon; Cyc A: Cyclosporine A; Cort: Cortisol; Sal: Saline

One of the shorter experimental time to produce a morphine dependent animal model, is to implant under the animals skin under a light anesthetic morphine pellet that contain 75 to 100 mg/kg morphine and after 72 hrs. to verify that the animal is morphine dependent to inject the morphine antagonist Nal and to observe the morphine withdrawal behaviors. This experimental procedure was used and presented in Figure 1. Seven behavioral expressions were recorded by two independent research associates from eleven experimental groups as follow from the left to the right side of the Figure 1: scream to touch, hyperactivity, exploratory behavior and diarrhea. These observations were rated as follow - no different from control = 0, and maximum activity = 5 (left side of Figure 1A and 1B). From the middle of the **Figure** to the right three behavioral expression were counted as follow: wet dog shake, teeth chattering and number of stools over 10 min after Nal (1.0 mg/kg) administration respectively [17,34]. Figure 1A green and Figure 1B red show significant (p<0.001) augmentation after Nal (1.0 mg/kg) administration in these seven behavioral expressions as compared to control group indicating the expression of behavioral withdrawal. Figure 1A in groups 4-violet, 5-blue and 6-orange were compared to the morphine depended group 3 green and show significant attenuation after Nal administration of the seven behavioral expressions of the withdrawal behaviors indicting that the immunomodifiers used attenuate the severity of morphine withdrawal. The result presented in the **Figure** demonstrate that the morphine elicit dependent by expression behavioral withdrawal (green column in Figure 1A and red column in Figure 1B), and each one of the three immunomodifiers IFN 150 IU/g, cyclosporine A (Cyc) 15 mg/kg, and cortisol (Cort) 2.0 mg/kg given prior to chronic morphine significantly attenuates (p<0.001) the expression of all the seven behavioral expressions of withdrawal signs (Figure 1A violet, blue and orange column) as compared to group 3 -green column.

Figure 1B summarizes similar experiment. The differences are that the immunomodifiers (IFN, Cyc or Cort) were administrated 72 hrs. after morphine pellet implantation i.e., the immunomodifiers were given to morphine depended animals, and one hr. later, Nal (1.0 mg/kg) was administrated. The observation indicates that treatment with each of the three immunomodifiers attenuates significant (p<0.001) severity of morphine withdrawal symptoms precipitated by Nal injection.

In conclusion: Figure 1A summarizes the above studies demonstrating that a single treatment of IFN, Cyc or Cort given prior to repetitive morphine exposure attenuate significantly (p<0.001) the expression of morphine withdrawal. IFN, Cyc or Cort given to morphine dependent animals (Figure 1) also attenuate significantly (p<0.001) the expression of morphine withdrawal symptoms precipitate by Nal exposure [17]. All of the above preclinical studies demonstrate that single treatment of immunotherapy can significantly attenuate morphine withdrawal symptoms and treat morphine dependent subjects and thus can prevent relapse and toxification. Similar observation was obtained in different morphine dependent experimental animal models when the morphine was given three times a day in escalating doses for five consecutive days to produce morphine dependent animal model [34–36]. How to explain this effect of the three immunomodifiers on morphine addiction is discussed in the next section.

## Discussion

**Figure 2:**
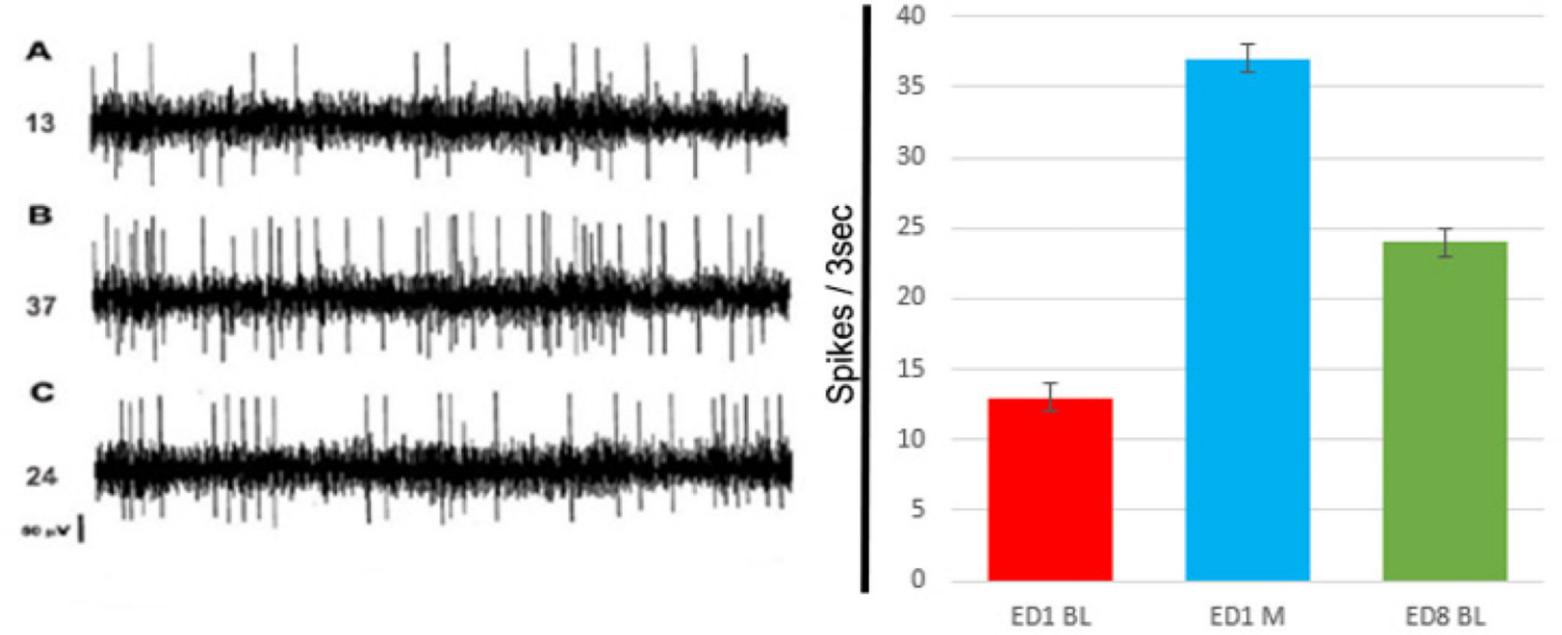
In the left side of the figure are three analog neuronal activity traces recorded from the prefrontal cortex (PFC) using Sprague Dawley male rats (Harlem Indianapolis IN USA). Each trace is three second of neuronal activities recording. In **(A)** experimental day 1 (ED1) is the activity following saline injection to provide baseline (BL) control. In **(B)** is the activity after 15 min post initial morphine (5.0 mg/kg) injection in ED1. The animals were treated the next 5 days repeatedly with the same morphine dose as at ED1 (5.0 mg/kg morphine) to elicit morphine dependency. In **(C)** is the activity 24 hrs. post last morphine suspension on ED8. The numbers 13, 37, and 24 in the left side of each trace are the total numbers of spikes activity of each of the 3 sec trace. In the right side of the figure is the histogram of the three analog traces of the neuronal activity showing in the left side of the figure demonstrating the BL at experimental day1 (ED1 BL) red histogram. The blue histogram summarizes trace B, showing the acute effect of 5.0 mg/kg morphine as compare to ED1 BL i.e., morphine elicits increase in neuronal activity as compare to ED1 BL – trace A compared to morphine ED1 the 2^nd^ trace. Additional 5 daily doses of morphine (5.0 mg/kg) were given at ED2 to ED6. Total six repetitive daily 5.0 mg/kg morphine were given followed by washout period and on ED8 (green histogram), i.e., ED8 BL activity demonstrating that after six daily morphine treatments and abrupt session of morphine the BL neuronal activity at ED8 as compare to ED1 BL was elevated, i.e., expression neurophysiological withdrawal activity.

Previous studies on the neurophysiologic properties underlying drug dependence have established that repetitive administration of morphine and other drugs of abuse modulate the behavioral and neuronal baseline (spontaneous) activities. These changes in spontaneous neuronal baseline activities in a large number of brain regions has been observed by us in 15 brain areas as follows: in the substantia nigra [37]; ventral tegmental area [38], locus coeruleus [38], dorsal raphe [39], nucleus accumbens [40], caudate nucleus [41], several thalamic [42] and several hypothalamic nuclei [42], reticular formation and central gray [37], hippocampus [42], amygdala [37], habenula [43], septum [37], and pre-frontal cortex [41]. These neurophysiologic changes correlate closely with the expression of tolerance and withdrawal and suggest a cause-effect relationship. When a drug of abuse is used repeatedly and withdrawn, it results in changes in the above brain structures’ neuronal baseline firing patterns (Figure 2). It is these electrophysiological changes that are causing the subsequent behaviors indicative of withdrawal [41,42]. We postulate that these electrophysiologic changes are causing the subsequent behavioral expression of withdrawal. Moreover, our previous electrophysiologic studies of the above 15 different brain structures demonstrate alterations in baseline neuronal activity following cession of the drug (Figure 2).

### IFN

Since the discovery of the endogenous opiate, the question that arises is: what prevents the development of tolerance of and/or dependence on these endogenous opiates? It was postulated that endogenous production of a protein or a peptide or immune system product such as a cytokine in the CNS could prevent the development of tolerance or physical dependence on these circulating endogenous opiates [28–30]. It was suggested that some endogenous substances are produced and released along with the endogenous opiates in order to prevent the organism from developing tolerance to or dependence on its own endogenous opiates [32]. Moreover, the endogenous level of IFN was reported to be reduced by repetitive morphine application [33]. The above was the rationale for a series of experiments testing if IFN has a direct effect on brain neuronal activity and if exogenous IFN will prevent and/or modulate the expression of opioid withdrawal.

Microiontophoretic (local) application of IFN, Nal, and 5HT on the single neuronal activity of 5 brain locations reverses the effect of morphine [44,45], suggesting that IFN, Nal, and 5HT modulate the morphine effects. Moreover, the endogenous level of IFN was reported to be reduced by repetitive morphine applications [32,33]. We hypothesized that application of IFN attenuates significantly and/or reverses the morphine withdrawal expression [34,46–50]. Alpha-interferon is an endogenous protein that possesses non-specific potent antiproliferation properties toward immunomodulatory activity. It rapidly produces a defense against foreign molecules [51,52] and is used for the treatment of chronic viral infections and malignant disorders [53]. Reyes-Vazquez et al., [33,45,53] reported that IFN modulates opiate-mediated phenomena by direct action on brain areas participating in the regulation of pain, temperature and food intake. Hori et al., [54] reported that IFN affects the central opiate receptors on neuronal membranes and on microglial cells, Reyes-Vazquez et al., [53] and Dougherty et al., [50] reported that in addition to opiate modulation, IFN modulates dopamine, serotonin and glutamate neurotransmitter systems as well endocrinologic and immunologic systems. Moreover, there is some structural similarity between IFN and endogenous endorphin [53,54].

Morphine consumption decreases the level of endogenous circulating IFN. Exogeneous IFN application modulate the immune system and leads to changes that reduce dopamine signaling and thus modifying the morphine effect that elicits dependency [55,56].

IFN stimulates cells to produce other proteins. IFN enhances the proliferation of human B cells and activates NK cells as well as dendritic cells that initiate immune responses through activation of inducible protein 10. This cytokine promotes Th 1 inflammatory response and is involved in signaling cells that affect the brain activity that controls craving and withdrawal behaviors [32]. IFN treatment was used in a clinical study on drug-addicted patients with chronic hepatitis and was successful in their treatment [53].

### Cyc A

Cyc A is a cyclic endecapeptide consisting of eleven amino acids and is a calcineurin inhibitors used as a powerful immunosuppressant medication [57]. Cyc A is a natural product from the fungus *Tolypoladium* [58]. Cyc A is known to decrease the production of nitric oxide, a mechanism through which it modulates morphine tolerance and dependency, modulates extracellular signal-regulated kinase (ERK) activation, and has a modulatory effect on cAMP which may be involved in signaling pathways in morphine-induced tolerance in cellular models [59]. Cyc A significantly decreases nitric oxide, and that decreases the chronic morphine-induced phosphorylation of ERK1/2 [60]. Dougherty and Dafny [35] measured brain Cyc A levels one hour after intraperitoneal injection of Cyc A. They reported that a significant level of the drug crossed the blood brain barrier and reached the brain to exert its effect directly on the CNS [49,61]. In a clinical study using Cyc A treatment in a patient treated with a stem cell transplant, the patient was treated with transdermal fentanyl to control pain. After fentanyl cessation, the patient developed severed behavioral withdrawal symptoms. When this patient was switched to repeated morphine dosing and several days later the morphine was discontinued, the patient did not develop the behavioral withdrawal symptoms [62]. This finding is consistent with previous pre-clinical studies [35,61]. Sanches-Covarrubias et al., [62] and Yang et al., [63] providing additional explanation how Cyc A exerts its effect on preventing the development of tolerance to morphine and the behavioral withdrawal symptoms. Cyc A induces p-glycoprotein which mediates morphine efflux and decreases morphine uptake thus preventing the development of tolerance and the expression of behavioral withdrawal. Additional explanations were provided by Yang et al. [63] regarding how Cyc A modulates opiate tolerance and behavioral withdrawal. Cyc A inhibits the transporter organic anion transporting polypeptide 2B1 (OATP2B1). OATP2B1 mediates blood-brain-barrier transport of morphine and the morphine-6-glucuronide (M6G) that is known to be a potent tolerance inducer. By inhibiting the intracellular accumulation of morphine and M6G and subsequent alteration of mu-opioid receptors and the calcium/calmodulin-dependent protein kinase 11 alpha expression and phosphorylation led to tolerance and withdrawal suppression. There is one publication [64] that reports that neither IFN nor Cyc A modulate the morphine withdrawal. This research group used a different experimental protocol and a different assay, and they concluded that these two immunomodifiers failed to attenuate the morphine withdrawal symptoms as several of the above studies reported.

### Cort

Cort is an endogenous end product of the hypothalamic-pituitary-axis (HPA) produced by the adrenal glands. Cort is a steroid hormone, one of the glucocorticoids, whose release is stimulated by adrenocorticotrophic hormone (ACTH). It is made in the outer cortex of the adrenal glands and then released into the bloodstream and transported throughout the body. It is responsible for regulating a wide range of physiologic processes [65]. Almost every cell contains receptors for Cort and thus Cort has many different actions depending on the tissue it is acting upon [66]. These effects include controlling glycemic levels involved in regulating metabolism, acting as an anti-inflammatory, influencing memory function, controlling salt and water balance, influencing blood pressure, helping with the development of the fetus [67,68], and influencing cognitive function [69–71]. Repetitive use of opiates induces effects on multiple levels of the endocrine system through mechanisms which are not fully elucidated [72]. Opiate use may induce endocrinopathies such as adrenal insufficiency due to suppression of the HPA [73–75], as well as functions of the brain motivational system [76] that may hold clues to the nature of the motivational changes accompanying addiction and vulnerability to addiction. Substance abuse causes stress-like responses. Repetitive consumption of opiates dysregulates the HPA and elicits deficient Cort activity that is associated with a risk for dependency and relapse after abrupt cessation of morphine [76]. Corticosteroids are nonspecific immunomodulators that modulate the immune system by affecting T-cells and B-cells, and thus suppress cell-mediated and humoral-mediated immunity [77]. In addition, macrophages and monocytes appear to be greatly affected by Cort. Activation of HPA arises from IL-1 stimulating hypothalamic corticotrophin releasing hormone thereby activating the ACTH in the pituitary which then activates the adrenal cortex to release glucocorticoids. This causes increase plasma concentrations of glucocorticoids and modulate the immune system [78–80]. Corticosteroids are nonspecific immunomodulators because they affect macrophages and monocytes as well as T-cells and B-cells, and hence cell-mediated and humoral-mediated immune processes [77]. Cort was also able to reduce significantly (P<0.001) the Nal-precipitated withdrawal syndrome [36]. Cort is anti-inflammatory acutely and pro-inflammatory in the long-term [81,82]. In humans, exogenous glucocorticoids are given to decrease inflammation and pain. It is likely that administration of cortisol before repetitive morphine provides negative feedback to the HPA preventing further release of glucocorticoids and, subsequently, immune modification. Morphine was reported to modulate the HPA axis [79] and Cort administration may restore the HPA axis. Opioids activate the downstream pathway of the HPA [83,84] suggesting that glucocorticoids modulate the immune system. Thus, Cort treatment may restore the HPA and reverse the effect of chronic opioid dependency and may explain why cortisol treatment attenuates the opiate withdrawal symptoms [36].

## References

[1] Vowles KE, McEntee ML, Julnes PS, Frohe T, Ney JP, Van Der Goes DN. Rates of opioid misuse, abuse, and addiction in chronic pain: A systematic Review and Data Synthesis. Pain. 2015 Apr 1;156(4):569–76. 25785523 10.1097/01.j.pain.0000460357.01998.f1

[2] Iqbal SM, Qamar I, Zhi C, Nida A, Aslam HM. Role of bisphosphonate therapy in patients with osteopenia: A Systemic Review. Cureus. 2019 Feb 27;11(2). 10.7759/cureus.4146 PMC6488345 31058029

[3] Krajcer Z, Ramaia LE h VG, Henao EA, Metzger DC, Nelson WK, Moursi MM Perioperative outcomes from the prospective multicenter Least Invasive Fast-Track EVAR (LIFE) registry. Journal of Endovascular Therapy. 2018 Feb;25(1):6–13. 29251207 10.1177/1526602817747871

[4] Pitt AL, Humphreys K, Brandeau ML. Modeling health benefits and harms of public policy responses to the US opioid epidemic. American journal of public health. 2018 Oct;108(10):1394–400. 30138057 10.2105/AJPH.2018.304590 PMC6137764

[5] Daniulaityte R, Juhascik MP, Strayer KE, Sizemore IE, Harshbarger KE, Antonides HM, Overdose deaths related to fentanyl and its analogs—Ohio, January–February 2017. MMWR. Morbidity and mortality weekly report. 2017 Sep 1;66(34):904. 28859050 10.15585/mmwr.mm6634a3 PMC5657791

[6] Welty L, Harrison A, Abram K, Olson N, Aaby D, McCoy K. Substance Abuse and Mental Health Services Administration.(2017). Key substance use and mental health indicators in the United States: Results from the 2016 National Survey on Drug Use and Health (HHS Publication No. SMA 17–5044, NSDUH Series H-52). Rockville, MD: Center for Behavioral Health Statistics and Quality. Substance Abuse and Mental Health Services Administration. Retrieved. College of Health Sciences. 2019 May;106(5):128.

[7] Caramello V, Bertuzzi L, Ricceri F, Albert U, Maina G, Boccuzzi A, The mass casualty incident in Turin, 2017: a case study of disaster responders’ mental health in an Italian level I hospital. Disaster medicine and public health preparedness. 2019 Dec;13(5–6):880–8. 31217041 10.1017/dmp.2019.2

[8] Dyer E, Swartzlander BJ, Gugliucci MR. Using virtual reality in medical education to teach empathy. Journal of the Medical Library Association: JMLA. 2018 Oct;106(4):498. 30271295 10.5195/jmla.2018.518 PMC6148621

[9] Green JM, Sundman MH, Chou YH. Opioid-induced microglia reactivity modulates opioid reward, analgesia, and behavior. Neuroscience & Biobehavioral Reviews. 2022 Jan 25:104544. 10.1016/j.neubiorev.2022.104544 PMC9699693 35090951

[10] Watkins LR, Hutchinson MR, Johnston IN, Maier SF. Glia: novel counter-regulators of opioid analgesia. Trends in Neurosciences. 2005 Dec 1;28(12):661–9. 16246435 10.1016/j.tins.2005.10.001

[11] Coller JK, Hutchinson MR. Implications of central immune signaling caused by drugs of abuse: mechanisms, mediators and new therapeutic approaches for prediction and treatment of drug dependence. Pharmacology & Therapeutics. 2012 May 1;134(2):219–45. 22316499 10.1016/j.pharmthera.2012.01.008

[12] Evans CJ, Cahill CM. Neurobiology of opioid dependence in creating addiction vulnerability. F1000Research. 2016;5. 10.12688/f1000research.8369.1 PMC4955026 27508068

[13] Achour SB, Pascual SO. Glia: the many ways to modulate synaptic plasticity. Neurochemistry international. 2010 Nov 1;57(4):440–5. 20193723 10.1016/j.neuint.2010.02.013

[14] Harricharan R, Abboussi O, Daniels WM. Addiction: A dysregulation of satiety and inflammatory processes. Progress in Brain Research. 2017 Jan 1;235:65–91. 29054292 10.1016/bs.pbr.2017.07.012

[15] Zhang H, Largent-Milnes TM, Vanderah TW. Glial neuroimmune signaling in opioid reward. Brain Research Bulletin. 2020 Feb 1;155:102–11. 31790721 10.1016/j.brainresbull.2019.11.012 PMC6946383

[16] Ihezie SA, Dafny N. Prevention of Opioid Addiction. Journal ISSN. 2021;2766:2276.

[17] Self WH, Tenforde MW, Rhoads JP, Gaglani M, Ginde AA, Douin DJ, 2021. Comparative effectiveness of Moderna, Pfizer-BioNTech, and Janssen (Johnson & Johnson) vaccines in preventing COVID-19 hospitalizations among adults without immunocompromising conditions—United States, March–August 2021. Morbidity and Mortality Weekly Report, 70(38), p.1337. 34555004 10.15585/mmwr.mm7038e1 PMC8459899

[18] Rogers TJ, Roy S. The Role of Opioid Receptors in Immune System Function. Frontiers in Immunology. 2021;12. 10.3389/fimmu.2021.832292 PMC8784803 35082800

[19] Re GF, Jia J, Xu Y, Zhang Z, Xie ZR, Kong D, Dynamics and correlations in multiplex immune profiling reveal persistent immune inflammation in male drug users after withdrawal. International Immunopharmacology. 2022 Jun 1;107:108696. 10.1016/j.intimp.2022.108696 35303506

[20] Dafny N. Evidence that the Immune System Participates in the Expression of Opiate Withdrawal Behavior. J Addict Sci S. 2020 Jun 8;6.

[21] Dafny N, Pellis NR. Evidence that opiate addiction is in part an immune response: destruction of the immune system by irradiation-altered opiate withdrawal. Neuropharmacology. 1986 Aug 1;25(8):815–8. 3774111 10.1016/0028-3908(86)90003-1

[22] Breehl N. The immune system: A new hope for opioid addiction? Labroots. 2018, https://www.labroots.com/trending/immunology/12679/immune-system-hope-opioid-addition [Accessed 03/2022].

[23] Liang X, Liu R, Chen C, Ji F, Li T. Opioid system modulates the immune function: a review. Translational perioperative and pain medicine. 2016;1(1):5. PMC4790459 26985446

[24] Xiaoyun S, Thomas KR. Immunotherapy for Drug Abuse. CNS Neurol Disord Drug Targets. 2011 Dec; 10(8): 876–879. 22229313 10.2174/187152711799219352 PMC3634568

[25] Xu A, Kosten TR. Current status of immunotherapies for addiction. Annals of the New York Academy of Sciences. 2021 Apr;1489(1):3–16. 32147860 10.1111/nyas.14329

[26] Zalewska-Kaszubska J. Is immunotherapy an opportunity for effective treatment of drug addiction?. Vaccine. 2015 Nov 27;33(48):6545–51. 26432911 10.1016/j.vaccine.2015.09.079

[27] Dafny N, Yang PB. Interferon and the central nervous system. European journal of pharmacology. 2005 Oct 31;523(1-3):1–5. 16226745 10.1016/j.ejphar.2005.08.029

[28] McCain HW, Lamster IB, Bozzone JM, Grbic JT. B-endorphin modulates human immune activity via non-opiate receptor mechanisms. Life Sciences. 1982 Oct 11;31(15):1619–24. 6292642 10.1016/0024-3205(82)90054-6

[29] Zimmermann E, Krivoy W. Antagonism between morphine and the polypeptides ACTH, ACTH1–24, and β-MSH in the nervous system. Progress in brain Research. 1973 Jan 1;39:383–94. 4363914 10.1016/S0079-6123(08)64094-7

[30] Bertolini A, Poggioli R, Fratta W. Withdrawal symptoms in morphine-dependent rats intracerebroventricularly injected with ACTH1–24 and with β-MSH. Life Sciences. 1981 Jul 20;29(3):249–52. 6270492 10.1016/0024-3205(81)90240-x

[31] Dafny N, Lincoln J. The role of interferons on the central nervous system in health and disease.

[32] Reyes-Vázquez C, Prieto-Gómez B, Dafny N. Interferon modulates central nervous system function. Brain research. 2012 Mar 9;1442:76–89. 22322149 10.1016/j.brainres.2011.09.061

[33] Dafny N. Interferon modifies morphine withdrawal phenomena in rodents. Neuropharmacology. 1983 May 1;22(5):647–51. 6192350 10.1016/0028-3908(83)90157-0

[34] Dougherty PM, Dafny N. Cyclosporine affects central nervous system opioid activity via direct and indirect means. Brain, behavior, and immunity. 1988 Sep 1;2(3):242–53. 3242657 10.1016/0889-1591(88)90026-8

[35] Montgomery SP, Dafny N. Cyclophosphamide and cortisol reduce the severity of morphine withdrawal. International journal of immunopharmacology. 1987 Jan 1;9(4):453–7. 3623770 10.1016/0192-0561(87)90019-1

[36] Dafny N, Marchand J, McClung R, Salamy J, Sands S, Wachtendorf H, Effects of morphine on sensory‐evoked responses recorded from central gray, reticular formation, thalamus, hypothalamus, limbic system, basal ganglia, dorsal raphe, locus ceruleus, and pineal body. Journal of Neuroscience Research. 1980;5(5):399–412. 7441794 10.1002/jnr.490050505

[37] Karim TJ, Reyes-Vazquez C, Dafny N. Comparison of the VTA and LC response to methylphenidate: a concomitant behavioral and neuronal study of adolescent male rats. Journal of Neurophysiology. 2017 Sep 1;118(3):1501–14. 28615331 10.1152/jn.00145.2017 PMC5596147

[38] Kharas N, Whitt H, Reyes-Vasquez C, Dafny N. Methylphenidate modulates dorsal raphe neuronal activity: Behavioral and neuronal recordings from adolescent rats. Brain Research Bulletin. 2017 Jan 1;128:48–57. 27889580 10.1016/j.brainresbull.2016.10.011 PMC5224521

[39] Claussen CM, Chong SL and Dafny N. (2014). Nucleus accumbens activity correlates to the animal’s behavioral response to acute and chronic methylphenidate. Physiol. & Behav, 129:85–94. 24534179 10.1016/j.physbeh.2014.02.024 PMC4116108

[40] Dafny N, Burks TF, Bergmann F. Dose effects of morphine on the spontaneous unit activity recorded from the thalamus, hypothalamus, septum, hippocampus, reticular formation, central gray, and caudate nucleus. Journal of Neuroscience Research. 1983;9(2):115–26. 6842623 10.1002/jnr.490090203

[41] Venkataraman SS, Claussen CM, Kharas N, Dafny N. The prefrontal cortex and the caudate nucleus respond conjointly to methylphenidate (Ritalin). Concomitant behavioral and neuronal recording study. Brain Research Bulletin. 2020 Apr 1;157:77–89. 31987926 10.1016/j.brainresbull.2019.10.009

[42] Dafny N. Multiunit recording from medial basal hypothalamus following acute and chronic morphine treatment. Brain Research. 1980 May. 10.1016/0006-8993(80)90304-2 7189436

[43] Dafny N, Qiao JT. Habenular neuron responses to noxious input are modified by dorsal raphe stimulation. Neurological Research. 1990 Jun 1;12(2):117–21. 1974700 10.1080/01616412.1990.11739929

[44] Reyes-Vazquez C, Prieto-Gomez B, Dafny N. Novel effects of interferon on the brain: microiontophoretic application and single cell recording in the rat. Neuroscience Letters. 1982 Dec 30;34(2):201–6. 6191255 10.1016/0304-3940(82)90176-8

[45] Reyes-Vazquez C, Prieto-Gomez B, Georgiades JA, Dafny N. Alpha and gamma interferons’ effects on cortical and hippocampal neurons: microiontophoretic application and single cell recording. International Journal of Neuroscience. 1984 Jan 1;25(1–2):113–21. 6084646 10.3109/00207458408985593

[46] Dafny N, Dougherty PM, Pellis NR. The immune system and opiate withdrawal. International journal of immunopharmacology. 1989 Jan 1;11(4):371–5. 2674032 10.1016/0192-0561(89)90083-0

[47] Dafny N. Is interferon-α a neuromodulator?. Brain research reviews. 1998 Mar 1;26(1):1–5. 9600621 10.1016/s0165-0173(97)00029-5

[48] Dafny N, Lee JR, Dougherty PM. Immune response products alter CNS acitivity: Interferon modulates central opioid function. Journal of neuroscience research. 1988 Jan;19(1):130–9. 2449543 10.1002/jnr.490190118

[49] Dougherty PM, Aronowski J, Drath D, Dafny N. Evidence of neuro-immunologic interactions: cyclosporine modifies opiate withdrawal by effects on the brain and immune components. Journal of Neuroimmunology. 1987 Jan 1;13(3):331–42. 3793881 10.1016/0165-5728(87)90068-3

[50] Dougherty PM, Harper C, Dafny N. The effect of alpha-interferon, cyclosporine A, and radiation-induced immune suppression on morphine-induced hypothermia and tolerance. Life Sciences. 1986 Dec 8;39(23):2191–7. 3784774 10.1016/0024-3205(86)90396-6

[51] Lengyel P. Biochemistry of interferons and their actions. Annual review of biochemistry. 1982 Jul;51(1):251–82. 10.1146/annurev.bi.51.070182.001343 6180680

[52] Pestka S, Baron S. [1] Definition and classification of the interferons. InMethods in enzymology 1981 Jan 1 (Vol. 78, pp. 3–14). Academic Press. 10.1016/0076-6879(81)78091-1 6173606

[53] Reyes-Vazquez C, Mendoza-Fernandez V, Herrera-Ruiz M, Dafny N. Interferon modulates glucose-sensitive neurons in the hypothalamus. Experimental brain research. 1997 Oct;116(3):519–24. 9372301 10.1007/pl00005780

[54] Hori T, Katafuchi T, Take S, Shimizu N, Niijima A. The autonomic nervous system as a communication channel between the brain and the immune system. Neuroimmunomodulation. 1995;2(4):203–15. 8963749 10.1159/000097198

[55] Capuron L, Pagnoni G, Drake DF, Woolwine BJ, Spivey JR, Crowe RJ, Dopaminergic mechanisms of reduced basal ganglia responses to hedonic reward during interferon alfa administration. Archives of general psychiatry. 2012 Oct 1;69(10):1044–53. 10.1001/archgenpsychiatry.2011.2094 PMC3640298 23026954

[56] Zhu JW, Liu FL, Mu D, Deng DY, Zheng YT. Heroin use is associated with lower levels of restriction factors and type I interferon expression and facilitates HIV-1 replication. Microbes and infection. 2017 Apr 1;19(4–5):288–94. 28104465 10.1016/j.micinf.2017.01.002

[57] Borel JF, Feurer C, Magnee C, Stähelin H. Effects of the new anti-lymphocytic peptide cyclosporin A in animals. Immunology. 1977 Jun;32(6):1017. 328380 PMC1445439

[58] Lafferty KJ, Borel JF, Hodgkin P. CYCLOSPORINE-A (CSA)-MODELS FOR THE MECHANISM OF ACTION. InTransplantation Proceedings 1983 Jan 1 (Vol. 15, No. 4, pp. 2242–2247). 655 AVENUE OF THE AMERICAS, NEW YORK, NY 10010: ELSEVIER SCIENCE INC.

[59] Rashki A, Mumtaz F, Jazayeri F, Shadboorestan A, Esmaeili J, Mehr SE Cyclosporin A attenuating morphine tolerance through inhibiting NO/ERK signaling pathway in human glioblastoma cell line: the involvement of calcineurin. EXCLI Journal. 2018;17:1137. 30713473 10.17179/excli2018-1693 PMC6341459

[60] Džoljić E, Grabatinić I, Kostić V. Why is nitric oxide important for our brain?. Functional Neurology. 2015 Jul;30(3):159. 10.11138/FNeur/2015.30.3.159 PMC4610750 26910176

[61] Dafny N, Wagle VG, Drath DB. Cyclosporine alters opiate withdrawal in rodents. Life Sciences. 1985 May 6;36(18):1721–6. 4039025 10.1016/0024-3205(85)90554-5

[62] Tsutsumi Y, Kanamori H, Tanaka J, Asaka M, Imamura M, Masauzi N. Withdrawal symptoms from transdermal fentanyl (TDF) after an allogeneic peripheral blood stem cell transplant (PBSCT). Pain Medicine. 2006 Mar 1;7(2):164–5. 10.1111/j.1526-4637.2006.00107.x 16634729

[63] Sanchez-Covarrubias L, Slosky LM, Thompson BJ, Davis TP, Ronaldson PT. Transporters at CNS barrier sites: obstacles or opportunities for drug delivery?. Current Ppharmaceutical Design. 2014;20(10):1422–1449. 10.2174/13816128113199990463 PMC3913737 23789948

[64] Yang ZZ, Li L, Wang L, Xu MC, An S, Jiang C, . siRNA capsulated brain- targeted nanoparticles specifically knock down OATP2B1 in mice: a mechanism for acute morphine tolerance suppression. Scientific reports. 2016 Sep 15;6(1):1–4. 10.1038/srep33338 PMC5024137 27629937

[65] Dantzer R, Phillips MI, Taylor EN, Gilman S. Dose effects of cortisol on single unit activity in hypothalamus, reticular formation and hippocampus of freely behaving rats correlated with plasma steroid levels. Brain Research. 1973 Sep 14;59:257–72. 4355885 10.1016/0006-8993(73)90265-5

[66] Andrews RC, Herlihy O, Livingstone DE, Andrew R, Walker BR. Abnormal cortisol metabolism and tissue sensitivity to cortisol in patients with glucose intolerance. The Journal of Clinical Endocrinology & Metabolism. 2002 Dec 1;87(12):5587–93. 12466357 10.1210/jc.2002-020048

[67] Hellhammer DH, Wüst S, Kudielka BM. Salivary cortisol as a biomarker in stress research. Psychoneuroendocrinology. 2009 Feb 1;34(2):163–71. 10.1016/j.psyneuen.2008.10.026 19095358

[68] Kudielka BM, Hellhammer DH, Wüst S. Why do we respond so differently? Reviewing determinants of human salivary cortisol responses to challenge. Psychoneuroendocrinology. 2009 Jan 1;34(1):2–18. 10.1016/j.psyneuen.2008.10.004 19041187

[69] Joseph JJ, Wang X, Spanakis E, Seeman T, Wand G, Needham B, . Diurnal salivary cortisol, glycemia and insulin resistance: The multiethnic study of Atherosclerosis. Psychoneuroendocrinology. 2015 Dec 1;62:327–35. 26356041 10.1016/j.psyneuen.2015.08.021 PMC4637243

[70] Kelly JJ, Mangos G, Williamson PM, Whitworth JA. Cortisol and hypertension. Clinical and Experimental Pharmacology and Physiology. 1998 Nov;25(S1):S51–6. 9809193 10.1111/j.1440-1681.1998.tb02301.x

[71] Law R, Clow A. Stress, the cortisol awakening response and cognitive function. International review of Neurobiology. 2020 Jan 1;150:187–217. 10.1016/bs.irn.2020.01.001 32204832

[72] Fountas A, Chai ST, Kourkouti C, Karavitaki N. Mechanisms of endocrinology: endocrinology of opioids. European journal of Endocrinology. 2018 Oct 1;179(4):R183–96. 30299887 10.1530/EJE-18-0270

[73] Donegan D. Opioid induced adrenal insufficiency: what is new?. Current Opinion in Endocrinology, Diabetes and Obesity. 2019 Jun 1;26(3):133–8. 10.1097/MED.0000000000000474 30870182

[74] Li T, Donegan D, Hooten WM, Bancos I. Clinical presentation and outcomes of opioid-induced adrenal insufficiency. Endocrine Practice. 2020 Nov 1;26(11):1291–7. 33471659 10.4158/EP-2020-0297

[75] Saeed ZI, Bancos I, Donegan D. Current knowledge and practices of heath care professionals on opioid-induced adrenal insufficiency. Endocrine Practice. 2019 Oct 1;25(10):1012–21. 31170362 10.4158/EP-2019-0177

[76] Lovallo WR. Cortisol secretion patterns in addiction and addiction risk. International Journal of Psychophysiology. 2006 Mar 1;59(3):195–202. 16434116 10.1016/j.ijpsycho.2005.10.007 PMC2257874

[77] Cupps TR, Fauci AS. Corticosteroid-mediated immunoregulation in man. Immunological reviews. 1982 Jan 1;65:133–55. 6214495 10.1111/j.1600-065x.1982.tb00431.x

[78] Al-Hashimi M, Scott SW, Thompson JP, Lambert DG. Opioids and immune modulation: more questions than answers. British journal of anaesthesia. 2013 Jul 1;111(1):80–8. 23794649 10.1093/bja/aet153

[79] Eisenstein TK. The role of opioid receptors in immune system function. Frontiers in Immunology. 2019 Dec 20;10:2904. 31921165 10.3389/fimmu.2019.02904 PMC6934131

[80] Ilies R, Judge TA. Goal regulation across time: the effects of feedback and affect. Journal of applied psychology. 2005 May;90(3):453. 15910142 10.1037/0021-9010.90.3.453

[81] Hofford RS, Russo SJ, Kiraly DD. Neuroimmune mechanisms of psychostimulant and opioid use disorders. European Journal of Neuroscience. 2019 Aug;50(3):2562–73. 30179286 10.1111/ejn.14143 PMC6531363

[82] Manetti L, Cavagnini F, Martino E, Ambrogio A. Effects of cocaine on the hypothalamic–pituitary–adrenal axis. Journal of Endocrinological Investigation. 2014 Aug;37(8):701–8. 24852417 10.1007/s40618-014-0091-8

[83] Ninković J, Roy S. Role of the mu-opioid receptor in opioid modulation of immune function. Amino acids. 2013 Jul;45(1):9–24. 22170499 10.1007/s00726-011-1163-0 PMC3912755

[84] Sacerdote P, Franchi S, Gerra G, Leccese V, Panerai AE, Somaini L. Buprenorphine and methadone maintenance treatment of heroin addicts preserves immune function. Brain, behavior, and immunity. 2008 May 1;22(4):606–13. 10.1016/j.bbi.2007.12.013 18294814

